# Investigating the association between grey matter hypoperfusion and atrophy in multiple sclerosis using arterial spin labelling

**DOI:** 10.1093/braincomms/fcag225

**Published:** 2026-06-24

**Authors:** Ilaria Boscolo Galazzo, Marco Castellaro, Francesca Benedetta Pizzini, Francesco Crescenzo, Michael Amann, Sarmad Al-Araji, Barbara Bellenberg, Alessia Bianchi, Frederik Barkhof, Rosa Cortese, Nicola De Stefano, Christian Enzinger, Massimo Filippi, Claudio Gasperini, Cristina Granziera, Ludwig Kappos, Carsten Lukas, Gloria Menegaz, Jacqueline Palace, Marco Pitteri, Maria A Rocca, Alex Rovira, Jaume Sastre-Garriga, Agnese Tamanti, Hugo Vrenken, Matthias Weigel, Marios C Yiannakas, Tarek Yousry, Olga Ciccarelli, Massimiliano Calabrese

**Affiliations:** Department of Engineering for Innovation Medicine, University of Verona, 37134 Verona, Italy; Department of Information Engineering, University of Padova, 35131 Padova, Italy; Padova Neuroscience Center, University of Padova, 35131 Padova, Italy; Department of Engineering for Innovation Medicine, University of Verona, 37134 Verona, Italy; Multiple Sclerosis Centre-Neurology Unit, ‘Mater Salutis’ Hospital, Azienda ULSS 9 Scaligera, 37122 Verona, Italy; Medical Image Analysis Center (MIAC) AG, 4054 Basel, Switzerland; Department of Neuroinflammation, Faculty of Brain Sciences, Queen Square MS Centre, UCL Queen Square Institute of Neurology, University College London, WC1N3BG London, UK; Ruhr University Bochum, St. Josef Hospital, Institute of Neuroradiology, 44791 Bochum, Germany; Department of Neuroinflammation, Faculty of Brain Sciences, Queen Square MS Centre, UCL Queen Square Institute of Neurology, University College London, WC1N3BG London, UK; Department of Neuroinflammation, Faculty of Brain Sciences, Queen Square MS Centre, UCL Queen Square Institute of Neurology, University College London, WC1N3BG London, UK; Department of Radiology & Nuclear Medicine, Amsterdam University Medical Center, Vrije Universiteit, 1081 Amsterdam, the Netherlands; Department of Neuroinflammation, Faculty of Brain Sciences, Queen Square MS Centre, UCL Queen Square Institute of Neurology, University College London, WC1N3BG London, UK; Department of Medicine, Surgery and Neuroscience, University of Siena, 53100 Siena, Italy; Department of Medicine, Surgery and Neuroscience, University of Siena, 53100 Siena, Italy; Department of Neurology, Medical University of Graz, 8036 Graz, Austria; Neuroimaging Research Unit, Division of Neuroscience, Istituto di Ricovero e Cura a Carattere Scientifico San Raffaele Scientific Institute, 20132 Milan, Italy; Neurology Unit, Istituto di Ricovero e Cura a Carattere Scientifico San Raffaele Scientific Institute, 20132 Milan, Italy; Vita-Salute San Raffaele University, 20132 Milan, Italy; Department of Neurosciences, S. Camillo Forlanini, Hospital Rome, 00152 Rome, Italy; Department of Biomedical Engineering, Faculty of Medicine, Translational Imaging in Neurology (ThINk) Basel, University Hospital Basel and University of Basel, 4123 Basel, Switzerland; Department of Neurology, University Hospital Basel, 4031 Basel, Switzerland; Research Center for Clinical Neuroimmunology and Neuroscience Basel (RC2NB), University Hospital Basel and University of Basel, 4031 Basel, Switzerland; Department of Biomedical Engineering, Faculty of Medicine, Translational Imaging in Neurology (ThINk) Basel, University Hospital Basel and University of Basel, 4123 Basel, Switzerland; Department of Neurology, University Hospital Basel, 4031 Basel, Switzerland; Research Center for Clinical Neuroimmunology and Neuroscience Basel (RC2NB), University Hospital Basel and University of Basel, 4031 Basel, Switzerland; Ruhr University Bochum, St. Josef Hospital, Institute of Neuroradiology, 44791 Bochum, Germany; Ruhr University Bochum, St. Josef Hospital, Department of Neurology, 44791 Bochum, Germany; Department of Engineering for Innovation Medicine, University of Verona, 37134 Verona, Italy; Department of Clinical Neurology, Nuffield Department of Clinical Neurosciences, University of Oxford, OX39DU Oxford, United Kingdom; Department of Neuroinflammation, Faculty of Brain Sciences, Queen Square MS Centre, UCL Queen Square Institute of Neurology, University College London, WC1N3BG London, UK; Department of Neuropsychology, National Hospital for Neurology and Neurosurgery, UCLH, WC1N3BG London, UK; Neuroimaging Research Unit, Division of Neuroscience, Istituto di Ricovero e Cura a Carattere Scientifico San Raffaele Scientific Institute, 20132 Milan, Italy; Neurology Unit, Istituto di Ricovero e Cura a Carattere Scientifico San Raffaele Scientific Institute, 20132 Milan, Italy; Vita-Salute San Raffaele University, 20132 Milan, Italy; Department of Radiology, Section of Neuroradiology, Hospital Universitari Vall d'Hebron, Universitat Autònoma de Barcelona, 08035 Barcelona, Spain; Centre d'Esclerosi Múltiple de Catalunya (Cemcat), Servei de Neurologia, Institut de Recerca Vall d'Hebron (VHIR), Hospital Universitari Vall d'Hebron, 08035 Barcelona, Spain; Department of Neurosciences and Biomedicine and Movement, The Multiple Sclerosis Center of University Hospital of Verona, 37134 Verona, Italy; Department of Radiology & Nuclear Medicine, Amsterdam University Medical Center, Vrije Universiteit, 1081 Amsterdam, the Netherlands; Department of Biomedical Engineering, Faculty of Medicine, Translational Imaging in Neurology (ThINk) Basel, University Hospital Basel and University of Basel, 4123 Basel, Switzerland; Department of Neurology, University Hospital Basel, 4031 Basel, Switzerland; Research Center for Clinical Neuroimmunology and Neuroscience Basel (RC2NB), University Hospital Basel and University of Basel, 4031 Basel, Switzerland; Division of Radiological Physics, Department of Radiology, University Hospital Basel, 4031 Basel, Switzerland; Department of Neuroinflammation, Faculty of Brain Sciences, Queen Square MS Centre, UCL Queen Square Institute of Neurology, University College London, WC1N3BG London, UK; Neuradiolological Academic Unit, BRR, UCL, WC1N3BG London, UK; Lysholm Department of Neuroradiology, UCLH, WC1N3GB London, UK; Department of Neuroinflammation, Faculty of Brain Sciences, Queen Square MS Centre, UCL Queen Square Institute of Neurology, University College London, WC1N3BG London, UK; National Institute for Health Research (NIHR), UCLH Biomedical Research Centre, NW12DA London, UK; Department of Neurosciences and Biomedicine and Movement, The Multiple Sclerosis Center of University Hospital of Verona, 37134 Verona, Italy

**Keywords:** perfusion, arterial spin labelling, MRI, multiple sclerosis, atrophy

## Abstract

The study of grey matter (GM) perfusion abnormalities in multiple sclerosis (MS) is gaining increasing interest, thanks to the recent developments in MRI techniques such as arterial spin labelling (ASL). Previous studies in small mixed cohorts have demonstrated reduced perfusion in different GM regions in patients with MS compared with controls, especially in the frontal/parietal lobes and deep GM. In this multi-centre study, we aimed to investigate the relationship between ASL-perfusion patterns and GM atrophy in a large cohort of MS patients. We hypothesized that GM hypoperfusion is a phenomenon not wholly associated with the presence of structural atrophy. Therefore, we have evaluated whether GM perfusion changes are directly explained by underlying atrophy or occur in the absence of detectable GM tissue loss. Moreover, the relationship between clinical (physical and cognitive) measures and global GM perfusion level was assessed. Conventional structural and pseudo-continuous ASL data were acquired using 3T MRI scanners and a standardized protocol in four European MS centres. We included in the analysis 170 subjects [96 relapsing-remitting MS (RRMS), 41 primary-progressive MS (PPMS) and 33 healthy controls (HC)] that exhibited acceptable data quality and compatible acquisition schemes/ASL maps. Global and regional cerebral blood flow (CBF) patterns were explored to identify group differences, statistically controlling for ‘morphometric covariates’ (i.e. total brain volume, cortical thickness or subcortical volume) along with age, sex, disease duration and total T2-lesion load in the analysis of covariance (‘basic covariates’). Similarly, partial correlation analyses were performed to assess the association of global CBF with physical (Expanded Disability Status Scale) and cognitive (Symbol Digit Modalities Test) scores. Global GM CBF values were lower in RRMS (64.1 ± 20.9 mL/100 g/min) and PPMS (52.9 ± 16.1 mL/100 g/min) compared with HC (78.5 ± 21.1 mL/100 g/min), with significant differences for both MS types compared with controls using both sets of covariates (p_Bonf_≤0.05). Moreover, regional perfusion reduction encompassing several cortical and subcortical GM regions were found in patients versus controls, even after the inclusion of the morphometric covariates, while there were no differences in GM perfusion levels between MS phenotypes. In all patients considered together, no significant associations between global GM CBF and physical disability/cognitive tests scores were found when controlling for the confounding factors. These findings suggest that GM hypoperfusion is at least partially dissociated with atrophy. In this scenario, ASL-based perfusion may be a promising functional paraclinical tool to quantify GM neurovascular impairment in MS, aiding the understanding of the pathological mechanisms.

## Teaser

Boscolo Galazzo *et al*. investigated the relation between grey matter patterns and arterial spin labelling (ASL)-based perfusion in multiple sclerosis (MS). Hypoperfusion over several cortical/subcortical regions in MS compared with controls was found, not often co-localized with significant structural changes. No significant differences in perfusion levels were depicted between the investigated MS phenotypes.

## Graphical Abstract

**Graphical Abstract:**
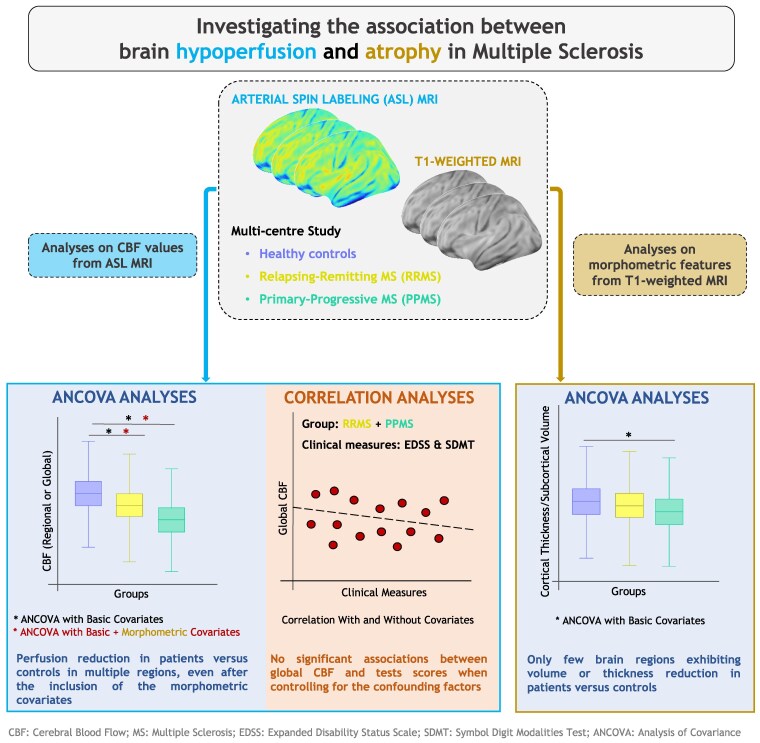

## Introduction

Multiple sclerosis (MS) is a chronic inflammatory demyelinating disease of the central nervous system characterized by parenchymal lymphocytic infiltration, focal demyelinated lesions and reactive astrogliosis in the white matter (WM), and meningeal inflammation, cortical demyelination and neurodegeneration in the grey matter (GM).^1^

Despite the growing knowledge about the disease, the pathogenetic mechanism(s) that lead to neurodegeneration are unclear. Cortical and deep GM atrophy, although most pronounced in the progressive phases, has been shown to be present from the earliest stages of the disease and has been suggested as a reliable predictor of long-term disability.^2-4^ Therefore, morphometric GM MRI-based measures represent sensitive and reproducible descriptors of (irreversible) neuronal and axonal damage in MS, albeit complementing such information with tissue microstructure or local functional properties can help to shed light onto the interplay between inflammation and neurodegeneration.

In this framework, interest in the potential contribution of neurovascular alteration to MS pathogenesis has grown. Previous studies employing nuclear medicine techniques in small cohorts of subjects with MS found decreased cerebral blood flow (CBF) affecting specific brain regions, mainly frontal GM areas, in primary progressive MS (PPMS) patients compared with relapsing-remitting MS (RRMS) and healthy controls (HC).^5^ More recently, MRI techniques based on contrast agents (dynamic susceptibility contrast (DSC) and dynamic contrast-enhanced) have been used to characterize perfusion patterns in MS patients demonstrating that local changes precede the blood-brain barrier breakdown and the WM lesion(s) appearance.^6,7^ Most of these contrast-based perfusion studies focused on WM lesions and normal-appearing WM (NAWM), suggesting that perfusion level changes in MS are dynamic over time and related to acute inflammatory processes.^8^ In particular, increased and decreased CBF was found in active and chronic WM lesions, respectively, compared with either perilesional NAWM in MS patients or WM in HC, along with generalized hypoperfusion in the NAWM of MS patients compared with normal WM of HC.^9-11^ When considering GM, decreased perfusion has been observed in subcortical structures (thalamus, putamen, and caudate) of MS patients, especially PPMS, compared with healthy controls.^12^

An important alternative method to inform about brain perfusion without the use of exogenous contrast agents (and possible associated risks) is represented by arterial spin labelling (ASL). ASL MRI uses magnetically labelled arterial blood water protons as an endogenous tracer for measuring tissue perfusion.^13^ For this reason, ASL has been increasingly used in clinical and research settings as a quantitative tool for assessing neuropathological processes.^14,15^ In MS, a few studies have exploited ASL sequences to measure CBF. Reduced CBF levels have been demonstrated in patients with MS compared with HC over GM and NAWM.^16-19^ When considering potential differences in perfusion across MS subtypes, only a few ASL studies at 1.5T are available.^20,21^ Rashid *et al*.^20^ in particular reported no regions of reduced perfusion in RRMS versus HC and RRMS versus PPMS, while brain-wide hypoperfusion encompassing cortical and deep GM structures has been demonstrated when comparing PPMS versus HC, especially striking in the thalamus and caudate nuclei.

Although the causes of GM-reduced perfusion in MS have not yet been clarified, it has been hypothesized that GM hypoperfusion may derive from neuronal loss and, consequently, lower energy demand.^8,22^ On the other hand, it has also been postulated that brain hypoperfusion precedes (or even induces) neuroaxonal damage in MS, possibly caused by the increased levels of vasospastic agents (i.e. endothelin-1) that have been demonstrated in patients with MS.^23,24^ Therefore, it became interesting to evaluate whether ASL might detect a reduction in GM perfusion without atrophy, which could indicate a reversible state of neuronal dysfunction before irreversible neuronal loss.^25-27^

ASL-based GM hypoperfusion in both cortical and deep GM has been demonstrated in the absence of atrophy early in the disease course of RRMS patients,^25^ further confirmed by another recent study illustrating how GM atrophy and hypoperfusion are evident in different brain areas at the same time, therefore not unequivocally linked.^26^ Moreover, Amann *et al*.^21^ demonstrated that T2-lesion load followed by age, sex and disease duration were the most significant factors in explaining cortical perfusion level reduction in MS patients (RRMS and secondary progressive MS), while cortical atrophy had a marginal impact on such measure. Thus, all these findings point to the absence of a direct association between perfusion and structural changes, at least in RRMS. At the same time, to the best of our knowledge, only limited findings on this aspect are currently available for PPMS, with a recent longitudinal study^28^ providing a detailed analysis of GM perfusion changes and their association with disability, complemented with the assessment of global/regional volume measures. Results demonstrated an overall decreased perfusion during time as well as atrophy over all regions during follow-up. Despite all these promising ASL findings, their interpretation is often hampered by several factors, such as the employed MRI technologies (mainly 1.5T scanners and outdated ASL sequences, not in line with the current guidelines^29^) and the limited sample sizes. Moreover, the perfusion-structural alterations link has been extensively investigated only in RRMS patients and relies on different statistical approaches, such as the correlation between GM CBF and atrophy measures, the separate voxel-wise analyses for depicting brain changes in patients compared with HC, or the inclusion of morphometry-related variables as covariates of no interest in the statistical models.

Therefore, in this study, we aimed to investigate both atrophy and perfusion patterns in a large cohort of MS patients (RRMS and PPMS) and HC to evaluate whether perfusion changes in GM occur in the absence of significant MRI-detectable thinning of the cortex or regional volume modifications and whether perfusion modifications are fully explained by underlying structural patterns. In addition, potential perfusion differences across MS phenotypes were explored to better characterize the corresponding pathological patterns. Lastly, the relationship between physical/neuropsychological assessments and global GM perfusion was assessed. We hypothesize that reduced GM perfusion would not be wholly associated with the presence of structural atrophy and would serve as a possible, clinically useful, marker of functional GM impairment in MS.

## Materials and methods

### Participants

In this study, subjects were prospectively recruited from April 2019 to June 2021 as part of a project assessing brain perfusion changes in MS at four European centres within the Magnetic Resonance in Multiple Sclerosis (MAGNIMS) Network (www.magnims.eu).

Eligibility criteria for RRMS and PPMS included: (i) a diagnosis of MS according to the revised 2017 McDonald Criteria^30^; (ii) no history of other neurological/psychiatric disorders or known non-MS brain lesions (of systemic inflammatory, vascular or traumatic origin); (iii) age between 18 and 65 years. In addition, age-matched HC with no neurological or psychiatric disorders were recruited in each centre. The total number of participants enrolled in the four sites was 199. The MAGNIMS network Steering Committee reviewed the final protocol for this study, and formal approval was received from the institutional review board at each committed centre. Each subject provided written informed consent. This cross-sectional study was reported in accordance with the STROBE guidelines.

### MRI acquisition

MRI acquisitions were performed on 3T scanners (Verona, Bochum, London: Philips Achieva, Best, the Netherlands; Basel: Siemens Prisma, Siemens Healthineers, Erlangen, Germany). ASL acquisition sequences were harmonized across scanners from different sites as best as possible and following the recommended guidelines,^29^ considering that the product ASL sequences fundamentally differ between vendors/scanners. The three Philips sites used an identical sequence based on 2D gradient-echo echo-planar imaging (EPI) pseudo-continuous ASL (2D EPI pCASL), while a 3D gradient- and spin-echo (3D GRASE) pCASL was implemented in the Siemens centre (Table 1). For the 2D EPI sequence, an additional calibration (M0) image was acquired with reversed phase-encoded blip polarity (A-P) for the subsequent estimation and correction of susceptibility-induced distortions.

**Table 1:** ASL acquisition parameters for both 2D and 3D sequences

	2D EPI (Verona, London, Bochum)	3D GRASE (Basel)
Scanner	3T Philips Achieva	3T Siemens Prisma
Labelling strategy	pCASL	pCASL
Labelling duration	1800 ms	1800 ms
Slice readout	36 ms	-
Post-labelling delay (PLD) (range)	1800–2484 ms	2000 ms
PLD (mean)	2142 ms	2000 ms
Labelling plane planning	90 mm below, parallel to ACPC line	90 mm below, parallel to ACPC line
Readout module	2D gradient-echo single shot, EPI with SENSE = 2.3	3D GRASE
Acquisition matrix	single-shot, 80 × 80	multi-shot, 80 × 80 (4 segments)
Number of slices	20	20
Slice thickness	5 mm	6 mm
Acquisition voxel size (volume)	3.0 × 3.0 × 5 mm (45 mm^3^)	3.0 × 3.0 × 6 mm (54 mm^3^)
Reconstruction voxel size	3.0 × 3.0 × 5 mm	3.0 × 3.0 × 6 mm
Slice gap	1 mm	n.a.
TR/TE	4330/12 ms	4010/16 ms
Number of signal averages	45	7
Background suppression (n pulses)	Yes (2)	Yes (2)
M0 sequence	Yes (TR = 10 s, no BS)	Yes (TR = 5 s, no BS)
Acquisition duration	06:38	04:10

High-resolution 3D structural images, including T1-weighted and T2-FLAIR, were also collected using the optimized local sequences (Supplementary Table 1).

### Clinical and neuropsychological assessment

Each MS patient underwent a complete neurological examination, including the Expanded Disability Status Scale (EDSS) assessment.^31^ Subsets of participants also supported additional data collection by participating in neuropsychological testing sessions performed at each participating site by experienced neuropsychologists. At the time of testing and data collection, the team consisted of experienced specialists, with over 5 years of experience in the field of MS. Across different neuropsychological tests, the Symbol Digit Modalities Test (SDMT) was considered. The SDMT was recognized as particularly sensitive to slowed information processing speed, commonly present in MS patients.^32^ Moreover, the SDMT was recently proven to be a sensitive measure of multiple cognitive processes, rendering it an optimal screening measure to detect altered cognitive functioning in MS patients.^33^

### MRI analysis

**Figure 1:**
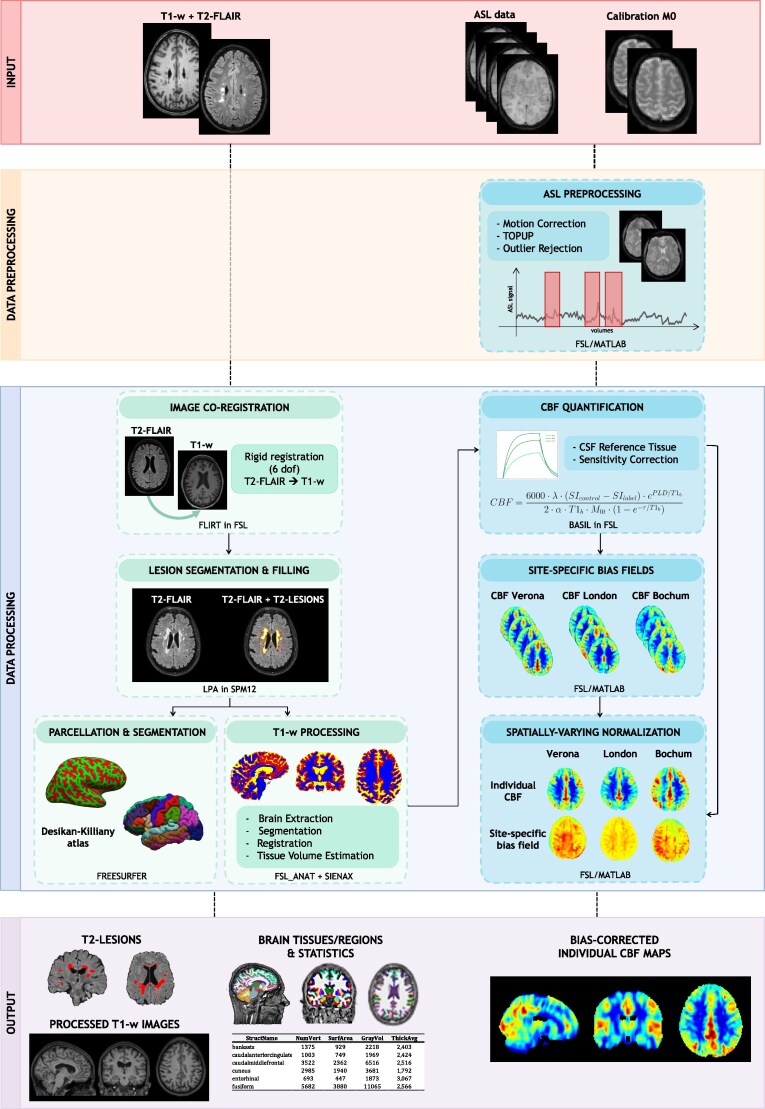
**Analysis worflow.** Schematic representation of the analysis pipelines for structural (T1-weighted images, left) and ASL data (right). T2-FLAIR images were coregistered to the corresponding T1-weighted scans and then fed to the LPA available in the LST for SPM12 to automatically segment the T2-hyperintense lesions and perform lesion filling. The resulting filled T1-weighted images were processed with FreeSurfer to derive a complete brain parcellation and segmentation. Moreover, they underwent anatomical processing with fsl_anat (in FSL) to perform registration and spatial normalization, among others, and SIENAX to estimate normalized total, grey and white matter volumes. Concerning ASL, after an initial data preprocessing (motion correction, topup and outlier rejection), CBF maps were quantified with the single compartment model, using the separate calibration scan (M0) and the output from fsl_anat as additional information. Once all these maps have been derived, a subsequent spatially-varying intensity normalization was applied to each map to adjust for possible site differences using site-specific bias fields estimated for each of the three centres (Verona, London, Bochum) in a multi-step procedure. This allowed to reduce the variability across different centres and mitigate its possible confounding effects, allowing to derive bias-corrected individual CBF maps to be used for all the subsequent analyses.

All MRI scans were anonymized and transferred to the referral MS centre of the University of Verona (Italy) that performed the analysis. A representative workflow of the whole analysis pipeline (structural and ASL data) is reported in Fig. 1. Of note, only the 2D pCASL data were included in the analyses for the present study to ensure the highest homogeneity possible. Indeed, despite the careful sequence harmonization across centres, we noticed blurring patterns and some macrovascular artefacts in the 3D GRASE data that were not present in the 2D data. Therefore, considering also the different readout and slice acquisition timing that could further increase the variability, we preferred to avoid any bias possibly introduced by these aspects by restricting the analyses to the 2D pCASL data.

#### Morphologic MRI analysis

T2-FLAIR images were rigidly registered for each patient to the corresponding T1-weighted scan using FLIRT.^34^ T2-hyperintense lesions were automatically segmented from T2-FLAIR images using the Lesion Prediction Algorithm (LPA) available in the Lesion Segmentation Toolbox (LST) for SPM12 (https://www.applied-statistics.de/lst.html).^35^ The generated lesion masks were further visually inspected and subsequently used to fill iso-hypointense focal areas in the corresponding T1-weighted images. In particular, the filling algorithm uses local information instead of global intensity distributions.^35^ The total T2-lesion load (volume) was also calculated for each patient.

Each individually filled T1-weighted image was then imported into the FreeSurfer software (v7.1.0, http://surfer.nmr.mgh.harvard.edu/, Harvard University, Boston, MA, USA) to perform a complete brain segmentation and parcellation (Desikan-Killiany atlas). Moreover, total brain volume (TBV), GM and WM volumes, all normalized for subject head size, were estimated with SIENAX^36^ in FSL 6.0.3. Finally, the FSL anatomical processing script (fsl_anat) was used to process each filled T1-weighted image, enabling, among the others, to perform bias-field correction, brain extraction and spatial normalization to standard space, using linear and non-linear registrations with FLIRT and FNIRT tools, respectively.

#### Perfusion MRI analysis

ASL data were preprocessed and analyzed using FSL and BASIL toolbox (https://asl-docs.readthedocs.io/en/latest/analysis_guide.html).^37^ Data were first realigned to account for spatial motion displacement and then corrected for distortion using the blip-reversed calibration images in TOPUP.^38^ An additional noise reduction step was applied, consisting of removing the outlier volumes as proposed by Tan *et al*.^39^

After the preprocessing steps, control-label difference images, along with the calibration scan and the fsl_anat output, were fed into BASIL to quantify CBF maps in absolute units (mL/100 g/min). The single compartment model^40^ with suggested relaxation times (tissue T1 = 1.3 s, arterial T1 = 1.65 s) and fixed labelling efficiency (α = 0.85) was used, while equilibrium blood magnetization was estimated using CSF as reference region. A subsequent voxel-wise correction was also applied to the estimated CBF maps to correct for possible sensitivity variation in the coil used for the acquisition, relying on the bias field map from fsl_anat. Perfusion images were then registered to the individual T1-weighted scan by using the boundary-based registration (BBR) cost function and further spatially normalized to the Montreal Neurological Institute (MNI) standard brain space with 2-mm spatial resolution, again using the previously estimated T1-weighted-to-MNI standard space (linear/non-linear) transformations.

Visual quality control was performed to check for major artefacts on CBF maps, mainly related to head motion, signal drop in the cerebral posterior regions, geometric distortions, and macrovascular bright spots.^41^ After excluding the worst quality scans, the remaining good CBF maps underwent a spatially-varying intensity normalization step as detailed in.^42^ Such an approach exploits the within-site CBF similarity between participants to remove the between-site quantification differences. It thus allows adjusting for site differences that could arise despite the sequence harmonization. This was done by considering the CBF maps in MNI space for each subject and deriving site-specific bias fields as follows: (i) a mean CBF image was created using the data from each site; (ii) this image was smoothed with a 6.4 mm full-width at half-maximum (FWHM) Gaussian kernel; (iii) these site-specific smoothed mean maps were averaged to create a population-average CBF, which was then rescaled to a mean GM CBF of 60 mL/100 g/min; (iv) site-specific bias-fields were created by dividing the population-average CBF image with the site-specific CBF image.

These site-specific bias fields were back-projected from MNI 2-mm to the individual T1-weighted using the previously estimated transformations and applied (i.e, multiplied) to correct the individual CBF maps, which were then used for all the subsequent analyses.

#### Regions of interest (ROIs)-based analysis

A subset of fifteen FreeSurfer-based brain regions, considered particularly relevant for the pathology based on literature,^43-45^ was retained for further analyses. In detail, six ROIs were selected from the individual subcortical segmentations that are thalamus (Thal), caudate (Cau), putamen (Put), and pallidum (Pall), representing deep GM nuclei, plus amygdala (Amy) and hippocampus (Hip, technically not subcortical but considered as such by FreeSurfer). In addition, the insula (Ins), precuneus (PCU), superior frontal gyrus (SFG), posterior cingulate cortex (PCC), peri calcarine (Perical), superior temporal gyrus (STG), superior parietal gyrus (SPG), precentral gyrus (PrG) and postcentral gyrus (PoG) were selected from the cortical parcellation. These 15 ROIs were used as masks to extract the regional CBF values from each subject. Moreover, a global GM CBF value was calculated for each subject using the individual probabilistic GM maps after thresholding at 0.8 and binarization.

Regarding morphometry, subcortical regional volumes (deep GM nuclei, Amy and Hip) and cortical thickness (CTh) values were obtained by FreeSurfer. All measures were averaged across the two hemispheres, and volume measures were further normalized by the estimated total intracranial volume.

### Statistical analysis

The differences between the regional CBF values were evaluated by performing an analysis of covariance (ANCOVA), separately for each brain region. In detail, each one-way ANCOVA having group as fixed factor was repeated twice, using at first a set of four ‘basic covariates’, that are age, sex, disease duration, and total T2-lesion load, and adding then to these variables the corresponding morphometry measure (i.e. normalized volume or CTh) as further confound. We hypothesize that ANCOVA with ‘morphometric covariates’ will highlight purer between-group perfusion differences, ensuring these are not driven only by the underlying GM atrophy. As each regional CBF measure was tested two times (ANCOVAs with and without morphometric measures), the raw *P*-value of the group factor was Bonferroni corrected (i.e. multiplied by a factor of 2) before performing the subsequent *post hoc* tests. *Post hoc* tests adjusted for multiple comparisons with Bonferroni correction were then computed when required.

ANCOVA was also applied to the global GM CBF values, using TBV as morphometric confound and the same multiple comparison corrections as above. As further analysis to verify whether global CBF changes in MS patients were possibly induced by the concomitant disease-modifying treatments (DMTs), one-way ANCOVA with basic and morphometric (TBV) covariates was applied to compare global GM CBF values between (i) treated versus untreated patients, and (ii) low/medium- versus high-efficacy treatments.

Similarly, the morphometry measures (regional subcortical volumes and CTh) were analyzed using only the four ‘basic covariates’ with one-way ANCOVA. Significant results were further investigated with adjusted *post hoc* tests (Bonferroni corrected).

Finally, a partial correlation analysis was performed to probe the association between global GM CBF values and physical (EDSS) and cognitive performance (SDMT) clinical scores. Non-parametric (Spearman) and parametric (Pearson) statistics were used for EDSS and SDMT, respectively. In detail, the four ‘basic’ and ‘basic + TBV’ variables were considered as covariates in the partial correlation analyses, in order to calculate the association between CBF and clinical scores while eliminating the influence of these other variables of no interest.

The significance threshold was set at *P* ≤ 0.05 for all statistical tests.

As an additional comparison, partial volume correction (PVC) was applied to the bias-corrected CBF maps to derive PVE-corrected maps subsequently used for deriving regional values and for performing ANCOVA analyses with ‘basic’ covariates. Details are reported in Supplementary materials.

## Results

### Demographic and clinical characteristics

177 subjects were enrolled in the three Philips sites (Verona, London, Bochum), while 22 were recruited in the single Siemens centre (Basel). As detailed above, the 3D GRASE data (9 RRMS, 3 PPMS and 10 HC) were not included in the analyses for the current study to ensure the highest homogeneity possible across the ASL images. While analyzing the 2D pCASL data, seven cases (3 RRMS, 2 PPMS and 2 HC) had to be discarded due to severe image artefacts that corrupted the perfusion estimates, leading to a final study cohort of 170 subjects. This included 137 patients (96 RRMS and 41 PPMS) and 33 HC, whose main demographic and clinical characteristics are summarized in Table 2. One hundred sixteen patients were under treatment with a disease-modifying drug: 47 were treated with low/medium-efficacy therapies (36 with dimethyl fumarate, 6 with teriflunomide, 5 with injectable immunomodulatory drugs as IFN beta or glatiramer acetate) while 69 patients with high-efficacy treatments (46 with ocrelizumab, 15 with cladribine, 6 with fingolimod, 2 with natalizumab). The remaining 21 patients were untreated.

**Table 2:** Main demographic and clinical characteristics, along with basic imaging descriptors, of healthy controls and patients with MS in the multi-centre study cohort (limited to the three Philips centres only)

	HC (*N* = 33)	RRMS (*N* = 96)	PPMS (*N* = 41)	*P*-value		
Age (y)	37.3 ± 9.5	41.7 ± 10.5	52.9 ± 8.1	<0.001^a^	HC versus RRMS	0.028
					HC versus PPMS	<0.001
					RRMS versus PPMS	<0.001
Sex (M/F)	15/18	30/66	22/19	0.036^b^	HC versus RRMS	ns
					HC versus PPMS	ns
					RRMS versus PPMS	0.013
Disease duration (y)		8.7 ± 6.4	11.9 ± 7.0	0.010^c^		
Total T2lesion load (mL)		5.8 ± 7.9	15.5 ± 16.3	<0.001^c^		
Median EDSS (range)		2.0 (0–7.0)	6.0 (1.0–7.0)	<0.001^d^		
Total brain volume (mL)	1526.1 ± 76.9	1464.1 ± 102.5	1392.4 ± 99.4		HC versus RRMS	0.002
				<0.001^a^	HC versus PPMS	<0.001
					RRMS versus PPMS	<0.001
Grey matter volume (mL)	808.3 ± 58.2	779.7 ± 63.9	726.7 ± 56.6		HC versus RRMS	0.022
				<0.001^a^	HC versus PPMS	<0.001
					RRMS versus PPMS	<0.001
White matter volume (mL)	717.8 ± 31.1	684.4 ± 48.0	665.7 ± 58.5		HC versus RRMS	0.001
				<0.001^a^	HC versus PPMS	<0.001
					RRMS versus PPMS	0.040

^a^Oneway analysis of variance + *post hoc* tests (uncorrected).

^b^Chisquare test + pairwise comparisons (uncorrected).

^c^Twosample *t*test.

^d^Wilcoxon ranksum test.

RRMS were significantly older than HC (*P* = 0.028), while PPMS patients were significantly older than both RRMS and HC (*P* < 0.001), and there was a significant difference in sex ratio between groups (RRMS versus PPMS, *P* = 0.013). Moreover, as expected, the two patient groups significantly differed in terms of disease duration, total T2-lesion load and EDSS (*P* < 0.05), with PPMS featuring higher values in all these characteristics. A significant difference in TBV was detected between groups (*P* < 0.001), with the lowest volume shown by PPMS. PPMS showed GM volume reductions compared with HC and RRMS (*P* < 0.001), and significantly lower GM volume values were also found in RRMS versus HC (*P* = 0.022). WM volumes revealed a similar pattern, with marked differences between HC and both RRMS/PPMS (*P* < 0.002) and *P* = 0.040 for RRMS versus PPMS.

For the sake of completeness, if Bonferroni correction is applied to account for the different between group comparisons (age, sex, three brain volumes variables), only a trend towards significance can be found for age and GM volume in HC versus RRMS, as well as for WM volume in RRMS versus PPMS.

Concerning the neuropsychological test (SDMT), this was acquired in a subset of 92/170 patients (59 RRMS and 33 PPMS). Median SDMT scores were 55 (range: 27–110) and 44 (range: 9–94) in RRMS and PPMS, respectively. These SDMT scores significantly differed between MS patient groups (*P* < 0.05).

### ASL perfusion results with and without morphometric covariates

**Figure 2:**
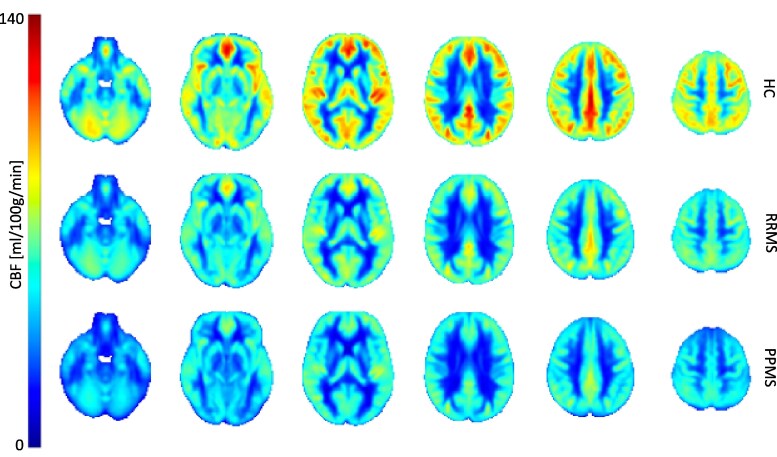
**Average CBF maps.** Bias-corrected mean CBF maps, expressed in mL/100 g/min, for the three groups. These were derived by averaging the individual maps in each separate group (*N* = 33 for HC, *N* = 96 for RRMS and *N* = 41 for PPMS).

The mean (bias-corrected) CBF maps for the three groups are illustrated in Fig. 2 for six representative slices. Their qualitative assessment revealed a marked generalized CBF decrease in the cortical and subcortical GM areas while moving from HC to PPMS patients. The global GM CBF values were 78.5 ± 21.1, 64.1 ± 20.9 and 52.9 ± 16.1 mL/100 g/min for HC, RRMS and PPMS, respectively.

One-way ANCOVA on these values including only the four ‘basic covariates’ confirmed the significant CBF decrease across groups (F(2163) = 4.35, *P* = 0.014). The significance of this result was further confirmed when including the additional specific fifth ‘morphometric covariate’ (represented by the TBV) in the analysis (F(2162) = 4.06, *P* = 0.019). Of note, such group differences remained significant even after the additional Bonferroni correction introduced for accounting for the two ANCOVA analyses applied on each CBF measure. With both sets of covariates, *post hoc* tests demonstrated significantly higher CBF values in HC compared with RRMS (p_Bonf_ = 0.022 for basic, p_Bonf_ = 0.022 for basic + TBV) and PPMS (p_Bonf_ = 0.022 for basic, p_Bonf_ = 0.041 for basic + TBV), while no significant differences were detected between RR- and PPMS.

There were no significant differences in global GM CBF values when comparing treated (85 RRMS and 31 PPMS) versus untreated (11 RRMS and 10 PPMS) patients (*P* > 0.05). Similarly, no differences between patients on low/medium- (44 RRMS and 3 PPMS) and high-efficacy treatments (41 RRMS and 28 PPMS) were found (*P* > 0.05). These findings were confirmed for both sets of covariates.

**Figure 3:**
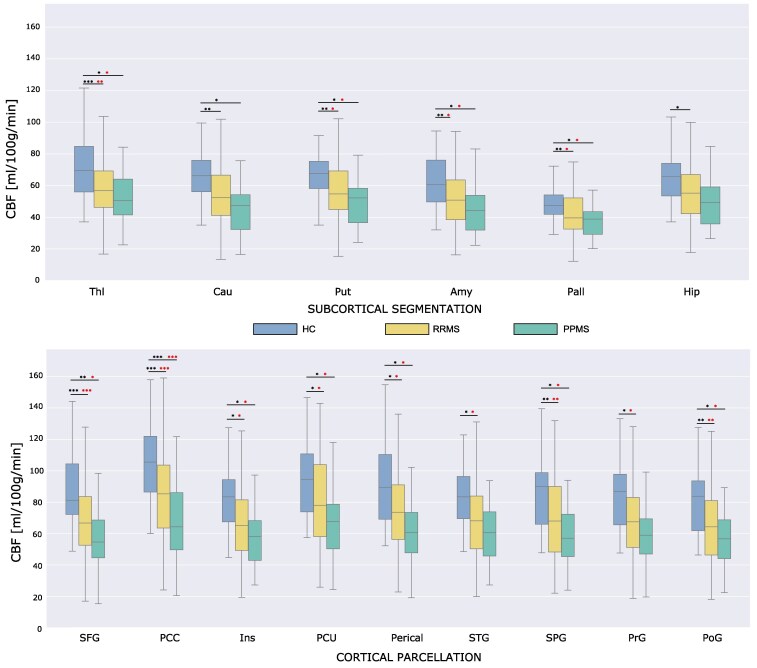
**Regional CBF values.** Distribution of bias-corrected regional CBF values in each of the three groups for the 15 regions of interest, including areas from the subcortical segmentation (top) and cortical parcellation (bottom). Black stars indicate significant *post hoc* results (p_Bonf_) from ANCOVA with basic covariates, while red stars refer to ANCOVA with basic + morphometric covariates. Boxplots denote the first and third quartiles, whiskers the min/max values excluding outliers and the black line the median value. * = p_Bonf_ ≤ 0.05; ** = p_Bonf_ ≤ 0.01; *** = p_Bonf_ ≤ 0.005. Amy, amygdala; Cau, caudate; Hip, hippocampus; Ins, insula, Pall, pallidum, PCC, posterior cingulate cortex; PCU, precuneus; Perical, peri calcarine; PoG, postcentral gyrus; PrG, precentral gyrus; Put, putamen; SFG, superior frontal gyrus; SPG, superior parietal gyrus; STF, superior temporal gyrus; Thal, thalamus. The total numbers were *N* = 33 for HC, *N* = 96 for RRMS and *N* = 41 for PPMS.

When looking at the results of the ROIs-based analyses, the distribution of the mean CBF values is reported in Fig. 3 for each region and group. Similar findings as above were found, with all one-way ANCOVA statistical analyses demonstrating significant between-group CBF differences (p_Bonf_ < 0.05) in the 15 regions when using the ‘basic’ covariates.

*Post hoc* tests resulted in significantly lower CBF values in all the 15 analyzed brain regions in RRMS compared with HC (p_Bonf_ ≤ 0.05), while three regions (Hip, STG and PrG) did not reach the significance when comparing PPMS versus HC (p_Bonf_ > 0.05).

Conversely, all the ROIs except Cau and Hip showed a significant main effect for the group variable when including the specific morphometric covariate, here represented by the corresponding regional normalized volume values, and applying the Bonferroni correction (p_Bonf_ < 0.05). For these 13 regions, the *post hoc* results were in line with those found with the ‘basic’ set of covariates, as reported in Fig. 3 and Supplementary Fig. S1. PCC was the region highlighting the most marked perfusion differences between MS patients and HC (p_Bonf_ ≤ 0.005) both with and without the ‘morphometric covariate’, followed by SFG and Thl. Other regions reporting between-group changes, especially for RRMS versus HC, were SPG, PoG, Put, Amy and Pall, reaching different significance levels.

Statistical results on PVE-corrected values were largely in line with those resulting from the main ANCOVA analyses (i.e. with and without morphometric covariates), showing control versus patient differences in PVE-corrected CBF values in almost all the regions (Supplementary material).

### Morphometric results

**Figure 4:**
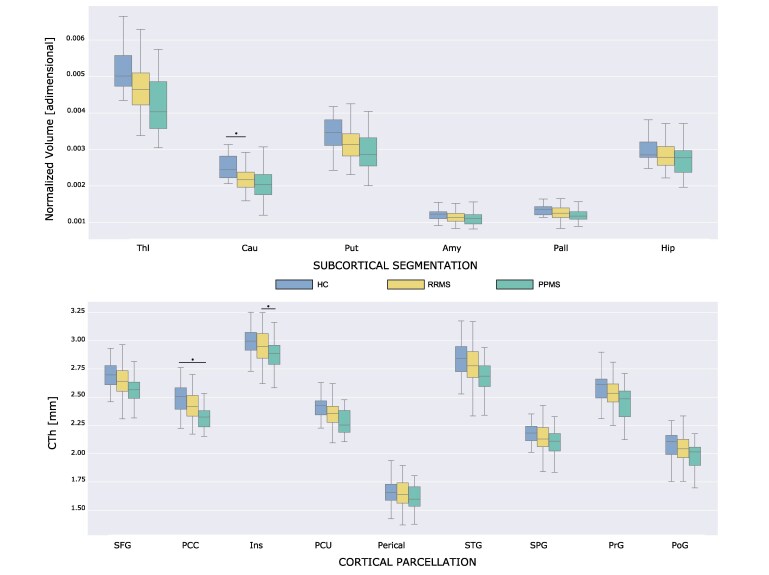
**Regional sMRI-based values.** Distribution of normalized volume (top) and cortical thickness (bottom) values for the regions from the subcortical segmentation and cortical parcellation, respectively, considered in the analyses, in each of the three groups. Significant *post hoc* results are indicated with a black star (p_Bonf_ ≤ 0.05). For interpretation of the boxplots, please refer to Fig. 3.

Different results were found when considering the morphometry measures (Fig. 4). In terms of regional volumes, only Cau showed a significant one-way ANCOVA (F(2163) = 3.67, *P* = 0.027) with *post hoc* tests highlighting significantly higher values in HC compared with RRMS patients (p_Bonf_ = 0.034). For CTh, ANCOVA analyses were significant in two regions only, that is PCC (F(2163) = 3.26, *P* = 0.041) and Ins (F(2163) = 3.11, *P* = 0.046). The former revealed higher thickness values in HC compared with PPMS at the *post hoc* tests (p_Bonf_ = 0.046), while for Ins a between-patients significant difference was found, with higher values in RRMS versus PPMS patients (p_Bonf_ = 0.045). All the remaining regions did not reach statistical significance in the ANCOVA analyses.

### Association of global GM perfusion levels with physical (EDSS) and neuropsychological (SDMT) tests scores

Partial correlation analyses including only ‘basic covariates’ revealed no significant associations between global GM CBF and EDSS or the SDMT (*P* > 0.05). These findings were confirmed when including the fifth ‘morphometric covariate’, represented by the TBV. However, significant results were found when no covariates were included in the analyses (EDSS: *r*_s_ = −0.296, *P* = 0.0004; SDMT: *r* = 0.359, *P* = 0.0004).

## Discussion

In this prospective, multi-centre study, we aimed at disentangling the possible relation between GM atrophy and perfusion alterations in a large cohort of MS patients (RRMS and PPMS), relying on a multi-parametric MRI approach to non-invasively measure regional subcortical volumes and CTh (T1-weighted structural MRI) and perfusion (ASL). Our findings demonstrated perfusion reduction encompassing several cortical and subcortical regions in patients with RRMS and PPMS compared with HC, not often co-localized with the significant structural changes and even after including the corresponding regional morphometric measure as covariate of no interest in the analyses. There were no significant differences in the perfusion levels between the two MS phenotypes. Moreover, no significant associations with either physical disability (EDSS) or cognitive status (SDMT) scores were found when controlling for several confounding factors in our patient cohort.

### Grey matter perfusion changes in MS

To characterize non-invasively the possible perfusion alterations occurring in such a MS cohort, we leveraged the potentialities of ASL. Although studying perfusion changes occurring in WM lesions and NAWM tissues might be hampered by the inherently low signal-to-noise ratio (SNR) and limited spatial resolution of this technique, ASL holds a great potential value when focusing on assessing the cerebral cortex and deep GM regions in MS.

Our findings revealed a global GM CBF reduction in MS patients compared with HC and identified some regions such as PCC, SFG and thalamus demonstrating reduced perfusion (especially in RRMS), largely in line with current literature results. Previous studies have indeed shown generally a global GM perfusion reduction in various cortical and subcortical areas when evaluating RRMS versus HC,^25,26,46^ with hypoperfusion over frontal/parietal regions, thalamus, and caudate consistently emerging when (mixed) MS patient groups were compared with HC.^16-18^ Of note, Debernard and colleagues reported contrasting results in two subsequent studies on RRMS and cortical/deep GM, firstly reporting hypoperfusion without atrophy^25^ and the opposite in a second one performed on a larger cohort.^47^ As recognized by the authors, such diverse findings could be related, among the others, to the different clinical characteristics of the enrolled subjects, thus requiring more detailed examinations and longitudinal studies to be further clarified.

We do not know the exact mechanism responsible for the CBF decrease in these regions. Regional reduced cortical perfusion could be due to a mechanism of retrograde trans-synaptic degeneration secondary to both severe damages in WM areas anatomically related to the hypoperfused GM regions or impairment of a specific neuronal pathway since the disease onset.^48,49^ In this scenario, this effect may depend on the number of cortical lesions, which may have altered CBF in the PCC and SFG, given that these regions have been shown to have a higher frequency of cortical lesions accrual over time.^50^ In addition, the damage to vasculature can result from the inflammatory insult to which some structures, such as the thalamus, are selectively subjected from the onset of the disease.^51,52^

Of note, decreased perfusion in cortical and deep GM of RRMS/PPMS has already been observed in different studies with other perfusion techniques besides ASL, further confirming the reliability of these results.^12,53^ One study with DSC-MRI also reported cortical/subcortical hypoperfusion in cognitively impaired patients with secondary-progressive MS relative to non-impaired patients, further supporting the presence of perfusion modulations in different MS types.^54^ However, it is worth mentioning an ASL study that reported opposite findings,^20^ with no regions of altered cerebral perfusion in RRMS versus HC, possibly due to the adopted ASL sequence (continuous ASL - CASL) and the lower magnetic field strength used (1.5T). The CASL sequence has some inherent limitations, including the long continuous radiofrequency (RF) pulse and the high specific absorption rate, discouraging its applicability in clinical settings. Conversely, the pCASL labelling here adopted provides high SNR, superior labelling efficiency and is compatible with modern body coil RF transmission hardware, being nowadays the recommended solution for clinical imaging.^29^

Despite these technical limitations and the smaller sample size compared with our study, Rashid *et al*.^20^ demonstrated the presence of prominent perfusion reductions in PPMS compared with controls in cortical/deep GM structures and adjacent WM areas, in line with our findings. Generally, data on GM perfusion in PPMS patients are very scarce, limiting proper comparisons. In this scenario, it is important to mention another longitudinal ASL study that, differently from ours and previous works, did not find significant perfusion differences over cortex and deep GM when comparing PPMS to HC. However, the estimated CBF values of the PPMS subjects were lower than those of the HC in all the regions considered in the study, as acknowledged by the authors, and a slight overall CBF decrease over time was described, potentially related to multiple factors such as neurodegeneration, reduced metabolism and maladaptive functional mechanisms.^28^ Of note, the clinical and demographic characteristics compared with our PPMS population are different, the HC group has a smaller sample size, the adopted ASL sequence is different (3D-GRASE PASL versus 2D-EPI pCASL) and parts of the processing steps are different due to, for example, the lack of an additional M0 scan that allows only the estimation of surrogate values and ‘rough’ CBF estimates, as reported by the authors. All these factors could contribute to partially explain such difference. Further studies focusing specifically on GM and PPMS are thus needed and important to clarify better how perfusion is changing in this MS phenotype.

When comparing GM perfusion in the two MS subtypes, our results showed no significant differences between RR- and PPMS patients when accounting for confounding variables, even the ‘basic’ set only. Significant CBF differences between MS phenotypes were detected only when no covariates were included in the statistical models (results not shown), meaning that the apparent difference in non-adjusted CBF can be explained fully by the differences in age, sex distribution, disease duration and total T2-lesion load. This is partly in line with literature where potential differences are predominately based on small numbers of subjects and have not been consistently demonstrated so far even with conventional perfusion techniques,^8^ with only a few studies available and reporting no differences between RRMS and PPMS^20^ or selective differences in NAWM, such as periventricular areas.^10^ Interestingly, Amann *et al*.^21^ described lower mean cortical GM CBF in secondary-progressive MS than in RRMS in a large MS cohort, but significance was lost after adjusting for factors such as age, sex, and disease duration and T2-lesion volume in line with our findings.

In support of this, it should be noted that the analysis on global GM CBF did not demonstrate significant differences both considering treated versus untreated patients and patients treated with low/medium- versus high-efficacy disease modifying drugs (for both sets of covariates), suggesting that the different treatment regimens seem not to have significant impact on global GM CBF.

### Relationship between grey matter perfusion and atrophy in MS

Since atrophy has been suggested as one of the possible causes of hypoperfusion in MS,^8^ several authors have evaluated the association between these measures in RRMS and HC, relying on different approaches. Conversely, a single work has deeply investigated ASL-based perfusion changes in a longitudinal study on PPMS, complementing it with the assessment of global/regional volume measures.^28^ Current literature on RRMS and HC has correlated GM atrophy (regional or global) and cerebral perfusion, finding no significant associations between these two measures,^55^ while other studies which applied voxel-wise approaches to investigate both volumetric and perfusion alterations^25,26^ generally reported hypoperfusion in the absence of atrophy. Furthermore, a novel multi-parametric MRI method was recently adopted to map relevant physiological parameters as the cerebral metabolic rate of oxygen consumption (CMRO_2_) across the cerebral cortex in a group of RRMS patients and controls, and compared such physiological estimates as a function of GM partial volumes between the groups. Their findings demonstrated that alterations in these parameters, including CBF and CMRO_2_, were robust to GM variations, suggesting that the group differences reflected aspects of proper tissue physiology without any potential confounding role of atrophy.^56^ Finally, a recent ROI-based study demonstrated widespread GM hypoperfusion in RRMS compared with HC in the presence of focal GM atrophy, with regional hypoperfusion that may possibly precede tissue loss in MS.^57^ Moreover, the authors described an association between GM hypoperfusion and irreversible WM damage. Interestingly, the spatial pattern of statistical CBF differences between RRMS and HC described by the authors was unchanged when including the region-wise GM volume as a covariate of no interest. In our study, a similar strategy was followed to shed light on such a link, in line with what has been done in a few previous papers,^27,57-59^ that is testing for any CBF difference while controlling for the possible confound effect of the underlying morphometry (volume or CTh). Despite its simplicity, this approach can provide relevant information about the perfusion/atrophy relation, which can be easily compared with what is currently available in the state of the art. Besides evaluating the global GM CBF, a set of fifteen ROIs that are known to be importantly affected by the disease were chosen.^44,45^ By comparing the statistical analyses on perfusion with and without volume/CTh (morphometric) covariates, we aimed to verify whether the perfusion levels differed between groups, accounting for any possible structural modification. Interestingly, 13 out of the 15 selected regions showed a significant group effect even after controlling for the morphometric covariate, meaning that such perfusion changes across groups cannot be fully explained by GM structure. *Post hoc* analyses confirmed that RRMS patients have lower perfusion values in these ROIs than controls, even after morphometry correction, and that this alteration holds for PPMS in several ROIs. Conversely, Cau and Hip showed only a trend towards significance in the between-group comparison, meaning that controlling for the subcortical volumes had a substantial impact and thus suggesting that a perfusion/atrophy dissociation might not exist in these areas.

In terms of morphometry pattern only, in this cohort of patients, we did not find a relevant effect of cortical atrophy as it has been previously reported several times, with different studies showing GM atrophy in regions associated with the limbic system and default mode network.^2,44,49^ However, a trend of reduced regional GM volume or cortical thickness in MS patients (more relevant in PPMS) is present and significant in a modest region subset, that is Cau, PCC and Ins. This is in line with the corresponding literature. In particular, the PCC has been demonstrated to be among the first regions to become atrophic in PPMS patients, while Cau and Ins were detected as showing early atrophy when considering mixed MS patients as a single group.^44^

PCC and Ins regions also exhibited perfusion reduction in both MS subtypes with respect to HC, suggesting that irreversible damage has occurred over these areas, while Cau did not show significant between-group differences when considering the additional morphometric measure in the ANCOVA analysis. However, the significance of the perfusion results after controlling for the corresponding morphometric covariate in the ANCOVA analysis for PCC and Ins confirms that perfusion changes occur at least partially independently of anatomical modifications and cannot be fully explained by possible differences in the underlying structure, as often postulated. This also holds for most of the other brain regions, where perfusion modulations were depicted even in the absence of relevant structural modifications, further strengthened by the GM correction in the statistical analyses and suggests that brain perfusion alterations might occur even in absence of significant GM atrophy in MS patients. Traditionally, GM neuronal loss has been widely considered as the key element determining the perfusion changes; the haemodynamic parameter is closely linked to total metabolic neuronal activity that is decreased when a neuronal loss is present. Conversely, our findings support a more recent hypothesis that describes hypoperfusion not as an epiphenomenon of neurodegeneration but as a multi-factorial (partially) independent pathological process.^8,28,60^ Although hypoperfusion’s cause was hypothesized to be related to retrograde hypometabolism due to axonal degeneration, no relationship has been found between hypoperfusion and impaired axonal or astrocytic metabolism,^16^ and axonal metabolic dysfunction cannot explain alone the decrease in CBF. Then, GM of MS patients suffers from a chronic inflammatory state, which could lead to vasodilation of venules and lower perfusion. Finally, the combined effect of transitory/acute perfusion changes during inflammatory disease activity and continuous/chronic inflammatory state over time (independent of acute activity) could lead to functional neuronal alterations even without fully-developed atrophy. The elevated levels of the vasospastic peptide endothelin-1 detected in MS patients could play an important role in explaining the perfusion changes observed in GM (not only in WM).^60-62^ The presence of detectable perfusion modifications in both RRMS and PPMS compared with controls irrespective of any GM atrophy (used as controlling nuisance effect) could potentially indicate a reversible state of neuronal dysfunction that could be captured earlier, before more severe and irreversible clinically eloquent damage.^25^ However, further longitudinal studies are serving to prove this hypothesis.

The preliminary results derived from the application of PVC and subsequent statistical comparison of the PVC-corrected GM and net CBF values for the different ROIs align with findings from the main analysis, supporting the idea that hypoperfusion changes are not totally driven by atrophy.

The application of PVC methods is still limited in the ASL field, possibly because the benefit of including this in the quantification pipeline is currently uncertain and a consensus on which approach to be employed is still not available.^63^ However, the combined reporting of corrected/uncorrected CBF images could help to move a step ahead to disentangle the perfusion/atrophy relation in MS, and thus should be further exploited in order to complement what derived so far with the simpler yet consolidated approaches.

### Relationship between grey matter perfusion, physical disability and cognitive performance

Despite converging evidence of perfusion alterations in MS, their relationship with clinical measures of physical disability and cognitive functions has not been established yet. Significant associations between perfusion measures and physical disability, composite functional scores, cognitive decline and fatigue have been demonstrated in several studies, though with conflicting findings in some cases.^10,12,19^ When considering ASL, no correlations have been reported between perfusion and physical/cognitive tests scores (e.g. EDSS, SDMT, Paced Auditory Serial Addition Test-PASAT) in most studies.^17,21,25,46,56^ Conversely, De la Pena *et al*.^18^ reported significant negative correlations between EDSS and perfusion in frontal regions and deep GM, while no correlations with SDMT were found. Areas of significant association with EDSS and/or SDMT, either positive or negative, were also reported by other authors,^27,28^ suggesting adaptive and maladaptive patterns. However, it is important to note that most of these previous perfusion studies reporting significant associations did not consider the possible confounding effect that different factors can induce. Similarly, we depicted significant positive (SDMT) and negative (EDSS) associations with global GM CBF when no covariates were included, which were nullified when controlling for the effects of the confounds, raising a note of caution on the possible spurious nature of such relationships. Further studies are thus needed to confirm these findings and to better clarify the relationship between GM perfusion and clinical/cognitive scores. Extending the set of physical and cognitive tests considered for such evaluations could also aid in disentangling their intricate relationship as EDSS and SDMT might be only partially appropriate in this scenario and might lack sensitivity for detecting significant associations with a physiological parameter as perfusion (e.g. about cognition, please see Pitteri *et al*.^64^).

### Technical considerations

From a technical point of view, our results proved the feasibility of performing an ASL multi-centre study in MS, though specific steps had to be taken as ASL perfusion analysis is very sensitive to the scanner acquisition protocol and sequence parameters.^29,42^ To cope with different sites and other subtle differences intrinsic in the data of each centre despite the sequence standardization, we further harmonized our combined dataset using a spatially-varying intensity normalization procedure proposed by Mutsaerts *et al*.^42^ to ensure that the data could be pulled together and mitigate possible scanner-related nuisance effects. In this first study, we decided to restrict the analyses to subjects scanned with the 2D pCASL sequence, considering the differences we noticed in the 3D GRASE data despite the careful sequence harmonization. This should ensure a more homogenous cohort and strengthen the results, avoiding possible biases related to the scanner/sequence types that might hide or mix the true perfusion patterns and thus increasing the confidence in the reliability of the results.

Importantly, we acknowledge that the global GM CBF values for HC are substantially higher than those typically reported in the literature. CBF values can vary significantly, even among healthy young adults, mainly due to intersubject/intrasubject variations, ASL sequence types and parameters, and processing pipelines. Typical absolute CBF values ranges from 50 to 70 mL/100 g/min in young and healthy adults in GM,^65^ though the ASL White Paper^29^ recommends, as a general rule, to consider GM CBF values varying from 40 to 100 mL/100 g/min as normal. This high variability often precludes drawing uniform conclusions across all the studies. Our CBF values are slightly higher compared with those reported in previous ASL/pCASL studies [for example in^57,66^], possibly influenced by some steps of the processing pipeline (e.g. quantification, spatially-varying intensity normalization step). We emphasize once again the need for caution when considering and comparing absolute (quantitative) CBF estimates across ASL studies, sequences and processing pipelines.

As further consideration of the study design, while our cross-sectional study allowed us to provide a picture of the GM CBF modulations occurring in MS and of the GM perfusion-atrophy relationship in a large cohort, longitudinal evaluations would further help in this respect. In particular, longitudinal studies on early MS are needed to better outline atrophy and perfusion changes and to clarify the possible perfusion role as early MRI-based functional marker. Moreover, there is a lack of large multi-centre and longitudinal studies employing standardized and harmonized measurement methods of perfusion and oxygen metabolic parameters that can further help the understanding of blood delivery alterations and, even better, disentangle them from neurodegeneration and atrophy.

Similarly, more advanced structural imaging techniques, such as diffusion MRI (dMRI), have the potential to provide quantitative measurements of microstructural changes in GM tissues in MS and thus help to further clarify the structure-function link. Some studies are present so far, either using classical diffusion tensor imaging^67^ or more advanced mathematical models,^45,68,69^ aiming at characterizing microstructural changes between controls and MS patients or between MS phenotypes. Widespread microstructural alterations and lack of overlap between the areas with changes in dMRI and volume have been generally described, especially at the beginning of the disease course. A few authors have also combined diffusion and perfusion metrics in GM, as in,^47^ though more studies are warranted to explore their mutual relationship and provide a complete characterization of structural/microstructural and perfusion modulations occurring over GM tissues in MS.

This picture could be further complemented with resting-state functional MRI (rs-fMRI) measures. Indeed, in the recent years, an increasing number of studies have explored rs-fMRI and in particular brain functional connectivity (FC) as possible tool to better understand the underlying mechanisms in MS, especially the cognitive symptoms.^70-72^ Complex patterns of regional increased/decreased FC in critical brain networks have been detected across different MS phenotypes as well as when comparing MS patients and controls, with some findings suggesting that MS pathology could differentially affect structural and functional networks throughout the course of the disease.^73^

Interestingly, a recent study focused on rs-fMRI to extract cerebral hemodynamics and oxygenation measures aiming at investigating differences in perfusion between WM lesions and normal-appearing brain regions in MS and HC, promoting this novel methodology as possible convenient disease-monitoring alternative to DSC-MRI and ASL.^74^

Finally, while an increasing number of studies is currently focusing on multi-modal MRI acquisitions in MS (as dMRI, fMRI, ASL^75,76^), further studies along this line are needed to better characterize the disease induced modulations (and their localization) and clarify the link between structural and functional network organization, despite the several technical, methodological, and biological challenges posed by the complex brain dynamics. Similarly, further insights on biological processes as inflammation and neurodegeneration can be derived from positron emission tomography (PET) with different radioligands. Going beyond those related to metabolism as [18F]-FDG, which has generally showed reduced metabolic values associated with clinical variables in MS patients, PET imaging based on the 18-kDa translocator protein (TSPO) is emerging as a valuable tool to characterize microglia activation in WM and GM. Moreover, imaging the colony stimulating factor 1 receptor (CSF1R) with tracers such as [^11^C]CPPC PET may offer a possible alternative for the specific (in-vivo) assessment of microglial activation and proliferation, although its applicability in MS remains to be investigated. Multimodal imaging approaches can thus help to further characterize the different modulations occurring in MS.^77-80^

## Conclusions

The relationship between perfusion and atrophy has been debated for a long time. With this prospective work, we add a further piece in this puzzle, using a non-invasive tool in a multi-centre approach and underpin the potential of ASL-based perfusion as a measure of neurovascular impairment in both RRMS and PPMS patients. Overall, our findings support the hypothesis of two processes of (i) neurodegeneration and GM loss (measured by atrophy) and (ii) neural metabolic dysfunction and inflammatory-stage-related alterations (measured by perfusion) acting at least partially independently and simultaneously in both MS phenotypes. ASL is a valuable tool deserving further evaluation, especially in PPMS and in longitudinal case-control studies, for better assaying its possible inclusion in clinical and research settings targeting the characterization of the haemodynamic status and functional organization in MS patients. Novel contrasts, such as the measurement of the CMRO2 along with the oxygen extraction fraction, may help better understand their relationship and the mechanisms of reduced GM perfusion.

## Supplementary Material

fcag225_Supplementary_Data

## Data Availability

The data that support the findings of this study are available from the corresponding author, upon reasonable request. The codes and tools (with their website) used in this study are reported in the Materials and methods section.
