# Supplementary material for: Investigating the association between grey matter hypoperfusion and atrophy in multiple sclerosis using arterial spin labelling
Source: Brain Commun. 2026 Jun 24;8(4):fcag225. doi: 10.1093/braincomms/fcag225 (PMC13487631; doi:10.1093/braincomms/fcag225)
Supplement: Supplementary file 1 [file fcag225_supplementary_data.docx]

**Supplementary Materials**

**Supplementary Table 1**

This reports the sequence type and the sequence parameters of both structural 3D T1-weighted and 3D FLAIR employed for each center. Regarding T1-w sequences, the centers used the same Turbo Field Echo readout except for Basel, which employed an MP2RAGE sequence that has two different readouts at two distinct inversion times accompanied with two different flip angles.

All 3D FLAIR sequences employed share a very similar readout, that is a Turbo Spin Echo with variable Flip Angles scheme implemented as BrainView or SPACE. We indicated that the sequence used a variable flip angles scheme, but not the values in the Flip angle 2.

|  | 3D T1-weighted | | | | 3D FLAIR | | | |
| --- | --- | --- | --- | --- | --- | --- | --- | --- |
| **Centre** | **Verona** | **London** | **Bochum** | **Basel** | **Verona** | **London** | **Bochum** | **Basel** |
| **Sequence type** | 3D-TFE | 3D-TFE | 3D-TFE | MP2RAGE | 3D-TSE-BrainView | 3D-TSE-BrainView | 3D-TSE-BrainView | SPACE |
| **Field of View** | 240x240 | 256x256 | 240x240 | 240x256 | 240x240 | 256x256 | 240x240 | 240x256 |
| **Slices** | 180 | 176 | 180 | 176 | 180 | 176 | 170 | 176 |
| **Voxel (mm)** | 1x1x1 | 1x1x1 | 1x1x1 | 1x1x1 | 1x1x1 | 1x1x1 | 1x1x1 | 1x1x1 |
| **TR (ms)** | 8.1 | 7 | 10 | 5000 | 8000 | 5000 | 4800 | 5000 |
| **TE (ms)** | 3.7 | 3.2 | 4.6 | 2.98 | 285 | 350 | 291 | 386 |
| **Flip angle 1 (deg)** | 8 | 8 | 8 | 4 | 90 | 90 | 90 | 90 |
| **Flip angle 2 (deg)** | - | - | - | 5 | <variable> | <variable> | <variable> | <variable> |
| **Inversion time 1 (ms)** | 900 | 823 | 1000 | 700 | 2356 | 1650 | 1650 | 1800 |
| **Inversion time 2 (ms)** | - | - | - | 2500 | - | - | - | - |
| **Averages** | 1 | 1 | 1 | 1 | 1 | 1 | 1 | 1 |
| **Total acquisition time (mm:ss)** | 04:51 | 06:35 | 05:44 | 08:20 | 05:02 | 06:35 | 06:57 | 05:40 |
| *Verona, London, Bochum: 3T Philips Achieva*  *Basel: 3T Siemens Prisma* | | | | | | | | |

**Supplementary Figure 1 – Statistical results on CBF measures.** Schematic representation of the 15 selected regions of interest, overlaid onto a T1-weighted image (top). Significant post-hoc results from the ANCOVA analyses with and without the inclusion of the corresponding morphometric covariates are also reported (bottom). Regions are color-coded according to the level of the corresponding p_Bonf_-values. For all the statistical comparisons between groups (controls vs patients with MS), perfusion values were higher in HC than in RRMS or PPMS. When including the morphometric covariates, two regions (Cau and Hipp) did not show a significant group factor, while a significance was still found for the remaining 13 regions of interest.

**Partial Volume Correction Analyses**

PVC was applied to the bias-corrected CBF maps using linear regression on a 5x5 kernel as described by Asllani and colleagues [2008] and as implemented in ExploreASL toolbox [Mutsaerts et al., 2020]. PVE-corrected CBF images for GM and WM were computed, along with PVE-corrected net CBF maps [Asllani et al, 2009; Borogovac et al, 2010; Petr et al, 2013; Ahlgren et al, 2014; Mutsaerts et al., 2020]. ANCOVA was finally applied to compare the regional values (GM CBF from PVE-corrected data and PVE-corrected net CBF) for the selected ROIs, using the “basic” covariates only.

Considering the PVE-corrected GM CBF values in the ROIs of the subcortical segmentation (Supplementary Figure 2), all regions showed HC vs RRMS statistical differences, while the HC vs PPMS comparison did not reach the significance in the Hip, as found in the main analyses, along with Cau and Put (a trend to statistical significance was though present).

Supplementary Figure 2 – PVC-corrected GM CBF values in each of the three groups (blue = HC, yellow = RRMS, green = PPMS) for the subcortical segmentations, reported as median values along with standard deviation across subjects. Stars indicate significant post-hoc results (pBonf) from ANCOVA with basic covariates * = pBonf ≤ 0.05; ** = pBonf ≤ 0.01; *** = pBonf ≤ 0.005.

For the cortical parcellation (Supplementary Figure 3), all regions showed HC vs RRMS statistical differences, although in some ROIs the statistical significance was reduced compared to the main ANCOVA analyses. The HC vs PPMS comparison did not reach the significance in the STG and PrG regions, in line with the previous findings. No differences were depicted also in this case between MS phenotypes.

Supplementary Figure 3 – PVC-corrected GM CBF values in each of the three groups (blue = HC, yellow = RRMS, green = PPMS) for the cortical parcellations, reported as median values along with standard deviation across subjects. Stars indicate significant post-hoc results (pBonf) from ANCOVA with basic covariates * = pBonf ≤ 0.05; ** = pBonf ≤ 0.01; *** = pBonf ≤ 0.005.

Results for the PVE-corrected net CBF values are also reported (Supplementary Figure 4 and Supplementary Figure 5 for subcortical segmentation and cortical parcellation, respectively), showing findings largely in line with the main ANCOVA analyses.

Supplementary Figure 4 – PVC-corrected net CBF values in each of the three groups (blue = HC, yellow = RRMS, green = PPMS) for the subcortical segmentations, reported as median values along with standard deviation across subjects. Stars indicate significant post-hoc results (pBonf) from ANCOVA with basic covariates * = pBonf ≤ 0.05; ** = pBonf ≤ 0.01; *** = pBonf ≤ 0.005.

Supplementary Figure 5 – PVC-corrected net CBF values in each of the three groups (blue = HC, yellow = RRMS, green = PPMS) for the cortical parcellations, reported as median values along with standard deviation across subjects. Stars indicate significant post-hoc results (pBonf) from ANCOVA with basic covariates * = pBonf ≤ 0.05; ** = pBonf ≤ 0.01; *** = pBonf ≤ 0.005.

**REFERENCES**

Ahlgren A, Wirestam R, Petersen E, Ståhlberg F, Knutsson L. Partial volume correction of brain perfusion estimates using the inherent signal data of time-resolved arterial spin labeling. NMR Biomed 2014; 27:1112-22

Asllani I, Borogovac A, Brown TR. Regression algorithm correcting for partial volume effects in arterial spin labeling MRI. Magn Reson Med 2008; 60:1362–71

Asllani I, Habeck C, Borogovac A, Brown T, Brickman A, Stern Y. Separating function from structure in perfusion imaging of the aging brain. Hum Brain Mapp 2009; 30:2927-35.

Borogovac A, Habeck C, Small SA, Asllani I Mapping Brain Function Using a 30-Day Interval between Baseline and Activation: A Novel Arterial Spin Labeling fMRI Approach. J Cereb. Blood Flow Metab 2010; 30: 1721–1733

Mutsaerts HJ, Petr J, Groot P, et al. ExploreASL: an image processing pipeline for multi-center ASL perfusion MRI studies. Neuroimage 2020; 219:117031

Petr J, Schramm G, Hofheinz F, Langner J, van den Hoff J. Partial volume correction in arterial spin labeling using a Look-Locker sequence. Magn. Reson. Med 2013; 70: 1535–1543
